# Left Atrial Myxoma Associated with Malignant Anomalous Course of Right Coronary Artery: A Rare Concurrent Incidence of Two Rare Diseases

**DOI:** 10.7759/cureus.4561

**Published:** 2019-04-29

**Authors:** George Yazigi, Kirolus Sourial, Sayed T Hussain, Patrick Mathias

**Affiliations:** 1 Internal Medicine, University of Central Florida College of Medicine, Orlando, USA; 2 Internal Medicine, Heart and Vascular Institute, Osceola Regional Medical Center/University of Central Florida College of Medicine/HCA GME Consortium, Kissimmee, USA

**Keywords:** cardiac tumor, left atrial myxoma, coronary artery anomalies

## Abstract

Cardiac atrial myxoma is a primary cardiac tumor with a prevalence of 0.0017-0.19% at autopsy. Anomalous origin of right coronary artery (ARCA) from opposite sinus of Valsalva is considered the second most common cause of sudden cardiac death among young athletes with an incidence of 0.09%-0.92% and mortality risk of 0-57%. We present a case of symptomatic left atrial myxoma in the presence of ARCA from the opposite sinus of valsalva. The myxoma was excised and the anomalous right coronary artery was re-implanted during the same operative session.

## Introduction

Myxomas are considered the most common primary cardiac tumors. Clinical presentation varies from being non-specific to a life-threatening presentation secondary to complications like stroke, heart failure, or sudden death [1].

Anomalous origin of right coronary artery (ARCA) from the left sinus of Valsalva has an incidence of 0.09%-0.92%, with a prevalence that is six times the one of anomalous origin of left coronary artery (ALCA). The mortality risk in ARCA is 0-57% compared to 30-100% in ALCA from sudden cardiac death [2, 3], and it is presumed to be the most common type of hemodynamically significant coronary anomaly [2]. Many patients with these anomalies are asymptomatic before the fatal event [3].

To our knowledge, this case is the first report of atrial myxoma associated with concurrent ARCA and where the patient underwent myxoma excision along with repositioning of the ARCA with re-implantation during the same open-heart surgery.

## Case presentation

**Figure 1:**
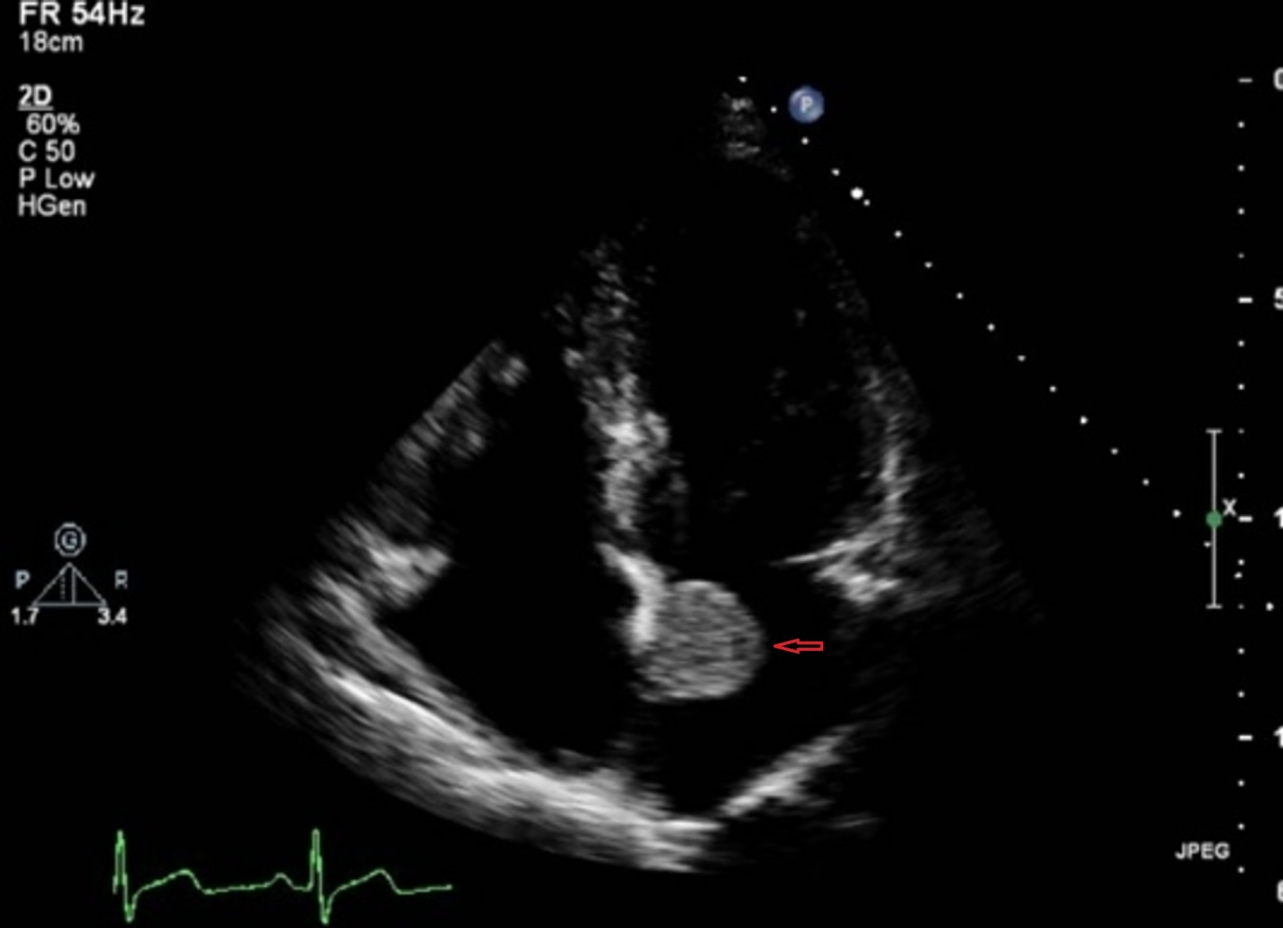
TTE frame shows left atrial myxoma. Red arrow points to left atrial myxoma. TTE: Trans-thoracic echocardiogram.

**Figure 2:**
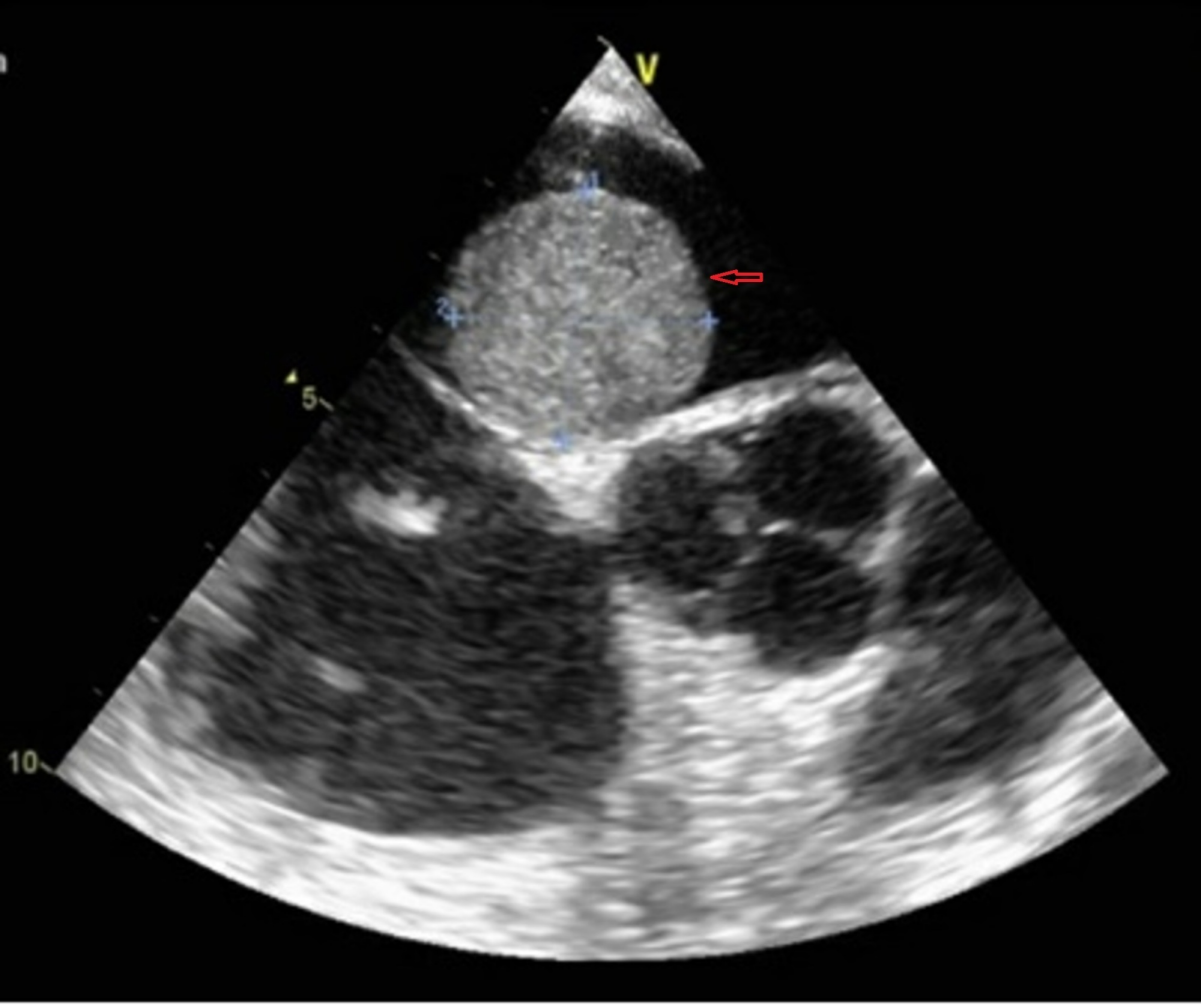
TEE frame shows left atrial myxoma. Red arrow points to left atrial myxoma. TEE: Trans-esophageal echocardiogram.

A 42-year-old African-American man, with a past medical history of pulmonary sarcoidosis and ARCA for which he was on conservative medical management, presented to the emergency department (ED) due to an episode of exertional presyncope which was relieved by rest. The patient denied any other associated symptom. Complete physical examination was unremarkable except for tachycardia with a heart rate of 106 beats per minute. Electrocardiogram (ECG) showed sinus tachycardia, left atrial enlargement and incomplete right bundle branch block without any dynamic ischemic changes. Cardiac enzymes including Troponin-I and Creatine Kinase-Muscle/Brain (CPK-MB) were negative. Trans-thoracic echocardiogram (TTE) (Figure 1) and trans-esophageal echocardiogram (TEE) (Figure 2) identified a 5.0 cm freely mobile left atrial echogenic mass suggestive of a left atrial myxoma attached to the interatrial septum and slightly going into the mitral plane. The new left atrial findings were not evident on a TTE that was done seven months earlier when the patient presented to the ED with an atypical chest pain.

**Figure 3:**
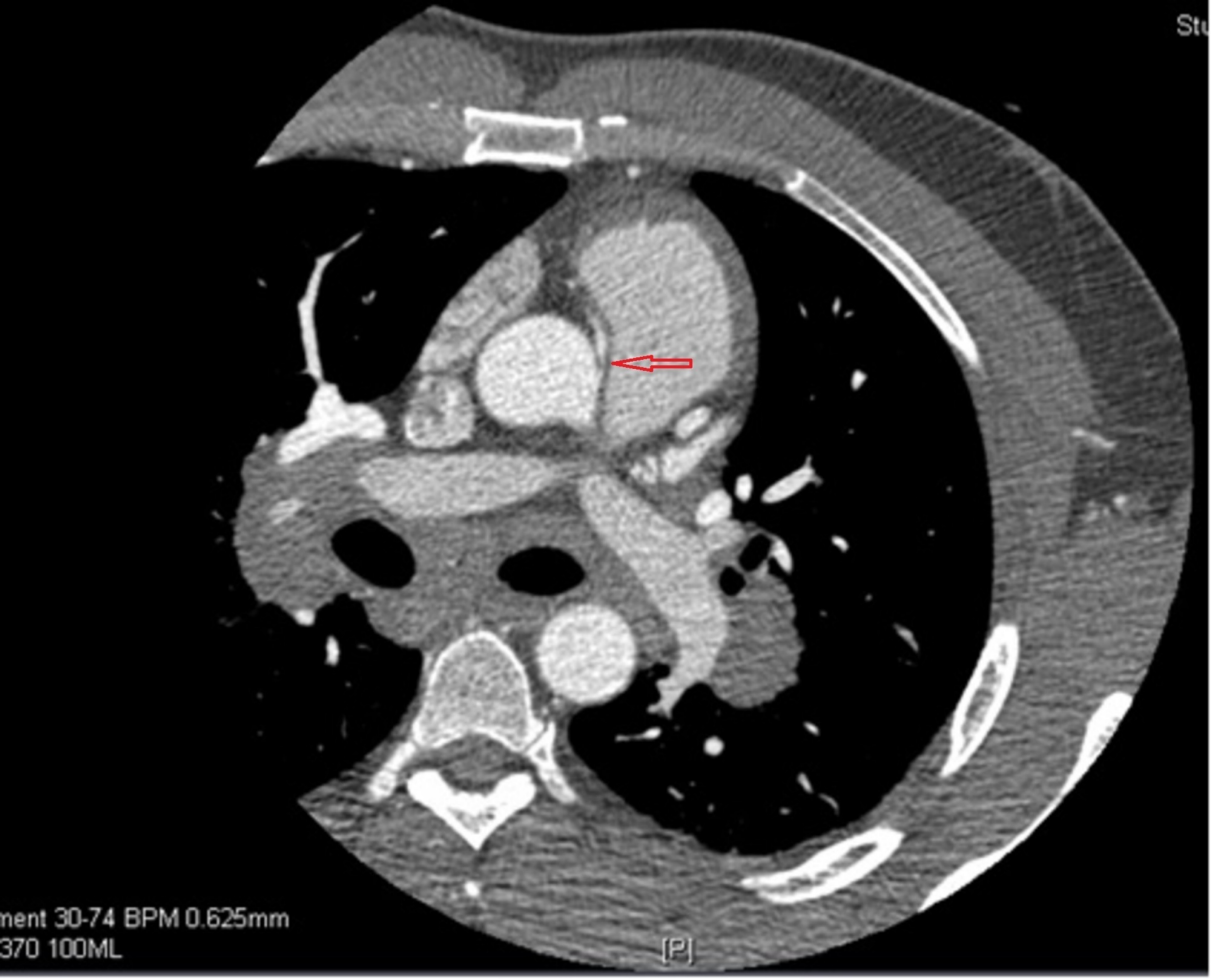
CCTA shows ARCA coming off the left coronary sinus, with slit like ostium (severe proximal right coronary artery narrowing). Red arrow points to ARCA. ARCA: Anomalous right coronary artery; CCTA: Coronary computed tomography angiography.

His ARCA was diagnosed by coronary computed tomography angiography (CCTA) (Figure 3) when he presented to the ED around 15 months earlier for recurrent atypical chest pain and exertional dyspnea. At that time a TTE was done and there were no signs of any cardiac tumor. His ARCA was found to have malignant course arising from the left coronary sinus superior to the left main coronary artery and coursing between the aorta and the pulmonary artery with diffusely narrowed proximal right coronary artery. A cardiac nuclear stress test was inconclusive. His symptoms were thought to be related to reactive airway disease and the decision was made for conservative medical management by avoidance of vigorous exercising.

The patient was offered a surgical excision of the left atrial mass. At this point, the patient preferred to also have his ARCA corrected during the same surgical session since he was aware of the risk of sudden cardiac death associated with this anomaly. He underwent surgical excision of the left atrial mass with repair of interatrial septum with fabric patch and transposition of the ARCA with re-implantation. Post-operatively the patient developed a complete heart block and he became ventricular pacemaker dependent. Otherwise, the patient tolerated the procedure well and was transferred to Cardio-Vascular Intensive Care Unit (CVICU) in a stable condition. Histopathological analysis of the excised mass confirmed the diagnosis of myxoma. A few days later, the patient was discharged in a good condition, remaining asymptomatic on a regular cardiology follow up for three years post-procedure.

## Discussion

Atrial myxoma manifestations depend on its anatomical location [1]. Typical manifestations include dyspnea and systemic embolization. Dyspnea presentation is thought to be due to intermittent mechanical atrioventricular obstruction and in severe cases can lead to pulmonary edema and heart failure symptoms [4]. In our case the patient presented with exertional presyncope, which also can be explained by the same mechanism leading to cardiac outflow obstruction. TTE is considered the initial diagnostic study for cardiac myxoma with a sensitivity up to 95% [5]. Surgical resection is the treatment of the choice [1].

Coronary artery anomalies arising from the opposite sinus of Valsalva are the second most common cause of sudden cardiac death among young athletes after hypertrophic cardiomyopathy [6].

Different features of ARCA have been reported. The anomaly is described depending on the course of its proximal part and its anatomical relation to the aorta and the pulmonary artery as: pre-aortic, inter-arterial (intramural or separated from the aortic wall), retro-cardiac, retro-aortic, intra-septal, and pre-cardiac (pre-pulmonary) [2]. The hemodynamically significant variant (i.e., inter-arterial course) is usually symptomatic or potentially dangerous. The optimal treatment remains controversial [2], and different treatment options vary depending on: the patient’s age, cardiovascular symptoms, percentage of proximal vessel narrowing, length of narrowing, and an intramural or inter-arterial course [7]. In our patient’s case the right coronary artery had a malignant course between the aorta and pulmonary artery but the patient’s cardiac stress test was negative.

Conservative medical management, including avoidance of vigorous exercise and beta blockers [8] to avoid exercise-induced ischemia and arrhythmias, is the most practiced management if no definitive signs of ischemia are seen [9], since the clinical outcome for ARCA is most often benign. This is in contrast to the justified management of all ALCA patients with surgical repair upon diagnosis [3].

Per 2008 AHA recommendations for ARCA, surgical coronary revascularization is recommended if there is ischemia secondary to inter-arterial course [2]. However, management of these coronary anomalies in asymptomatic patients is still less well defined, creating a risk-benefit dilemma [3]. Unique to this patient is the coincidence of the atrial myxoma with ARCA which has not been described before.

The patient was stable on conservative medical management trial, knowing that the incidence of sudden death in asymptomatic patients with ARCA is extremely low [3]. One review showed that among 6,300,000 military recruits who were screened for sudden death, 126 deaths were nontraumatic, 86% of which were exercise related. No patient had a right coronary artery (RCA) arising from the left coronary artery (LCA) sinus and in 21 the autopsy findings were indicative of an LCA arising from RCA sinus [10]. Our patient’s preference was to have his anomaly corrected during the myxoma resection open heart surgery. Therefore, a decision was made to do repositioning surgery for his ARCA along with the myxoma resection. Such an indication is not described previously in the literature due to the rarity of this co-incidence. An interesting question is whether patients with this anomaly benefit from surgical correction of ARCA if they are going to have open heart surgery for another indication.

## Conclusions

Surgical management of ARCA has a low risk of perioperative mortality but the long-term survival needs further study to clarify whether and when to intervene in an asymptomatic patient with an ARCA from the left coronary sinus.
